# Superior Gluteal Artery Bleed After Cephalomedullary Nail Fixation

**DOI:** 10.31486/toj.19.0084

**Published:** 2020

**Authors:** Colin Carroll, Michael Warren, Michael Nammour, Heather Taillac, James Mautner

**Affiliations:** ^1^ The University of Queensland Faculty of Medicine, Ochsner Clinical School, New Orleans, LA; ^2^ Department of Orthopedics, Ochsner Clinic Foundation, New Orleans, LA

**Keywords:** *Anemia*, *bone nails*, *embolization–therapeutic*, *hip fractures*, *postoperative hemorrhage*

## Abstract

**Background:** Hip fracture is a common orthopedic condition that leads to many hospitalizations each year. Intertrochanteric femur fractures are commonly treated with cephalomedullary nail fixation. Superior gluteal artery bleed is a rare complication of cephalomedullary nail fixation, especially when the trochanteric approach is used.

**Case Report:** A 63-year-old male presented to the emergency department with a right intertrochanteric femur fracture after a fall from standing height. Cephalomedullary nail fixation was performed without any complications during the operation. The patient's postoperative course was complicated by decreasing hemoglobin levels despite blood transfusions. Superior gluteal artery bleed with a large hematoma was discovered on postoperative day 4. The bleed was embolized, and the patient was stabilized and discharged.

**Conclusion:** We found only 1 published report of a superior gluteal artery bleed associated with nail placement. During the operative procedure, guidewire placement requires careful consideration because of the risk of vascular damage. Superior gluteal artery injury, although rare, should be considered in patients with unstable hemoglobin levels after nail placement.

## INTRODUCTION

Hip fracture is a common orthopedic injury that leads to the hospitalization of approximately 300,000 patients in the United States each year.^1,2^ A significant proportion of these hip fractures are intertrochanteric or pertrochanteric fractures, accounting for approximately 150,000 cases per year.^1,3-5^ Most surgeons prefer to use a cephalomedullary nail for intertrochanteric femur fractures instead of a sliding hip screw because of the ease of surgical technique, as well as better stability for fractures that are deemed unstable.^6^ Complications of cephalomedullary nail fixation include nonunion, malunion, periprosthetic fracture, and bleeding.^7,8^ Superior gluteal artery injury during cephalomedullary nail placement is rare and scarcely documented in the literature.^9^

We present a case of a superior gluteal artery bleed after cephalomedullary nail fixation for an intertrochanteric femur fracture.

## CASE REPORT

A 63-year-old male with a history of alcohol abuse presented to the orthopedic department for routine 6-week postoperative follow-up after surgical repair of a left hip fracture. The patient's left intertrochanteric fracture had been treated with cephalomedullary nail fixation without complication. At the follow-up appointment, the patient mentioned that he had recently fallen onto his right hip and had severe right hip pain. He was sent to the emergency department for evaluation.

**Figure 1.**
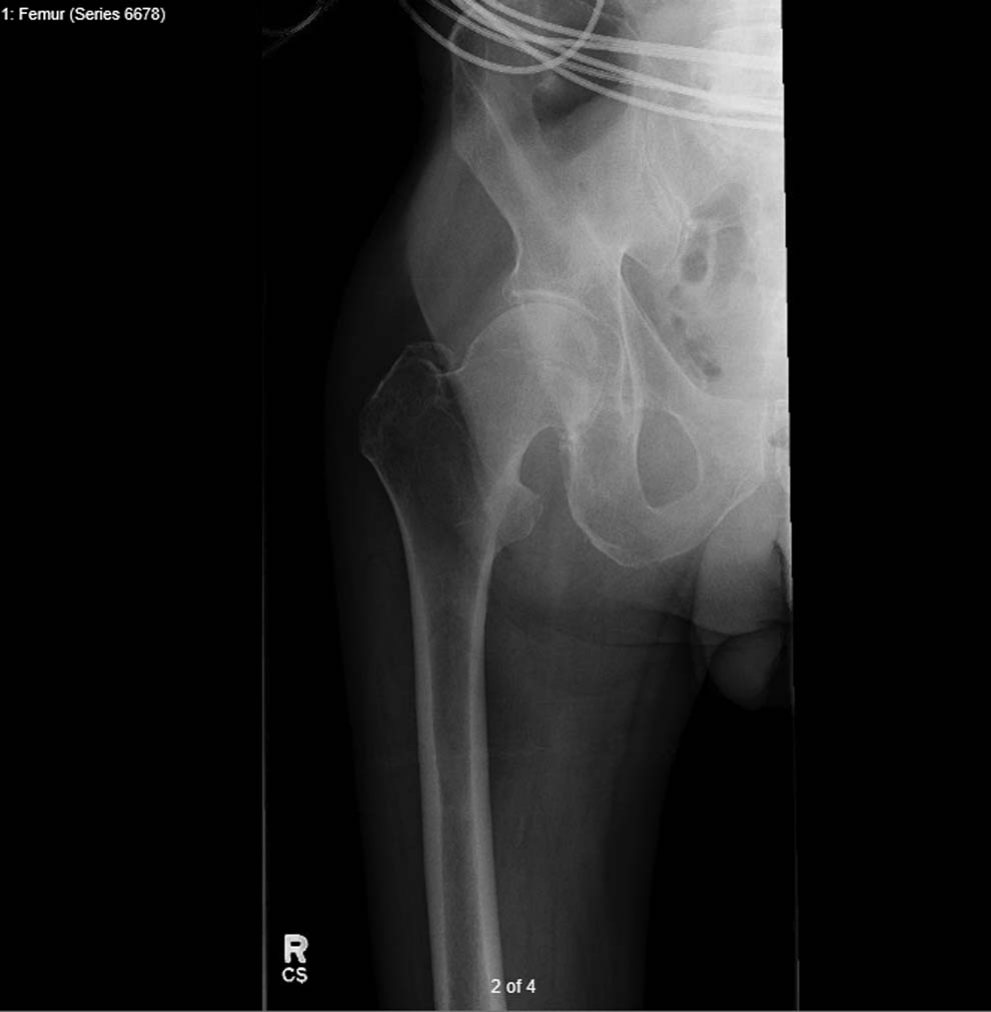
**Anteroposterior x-ray of the right femur shows a possible greater trochanteric chip fracture.**

**Figure 2.**
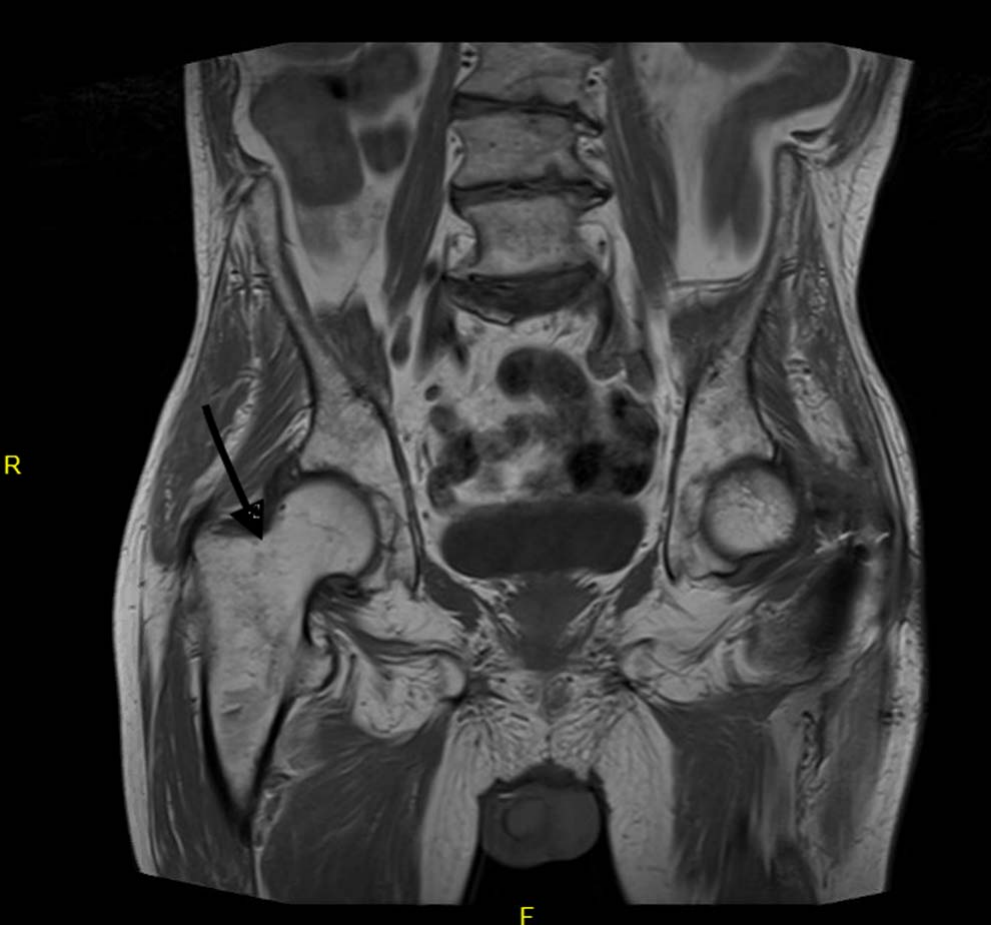
**T1 magnetic resonance imaging of the pelvis shows a right greater trochanteric fracture with extension into the lesser trochanter.**

Anteroposterior (AP) pelvis and right femur plain films showed a possible acute right greater trochanteric chip fracture (Figure 1). T1 and T2 magnetic resonance imaging sequences showed a fracture line extending from the greater trochanter to the lesser trochanter with surrounding edema, suggesting an intertrochanteric fracture (Figure 2). Ultrasound of the lower extremities revealed a partially occlusive deep vein thrombosis in the left common femoral vein. The treatment decision was Greenfield filter placement and right cephalomedullary nail fixation.

**Figure 3.**
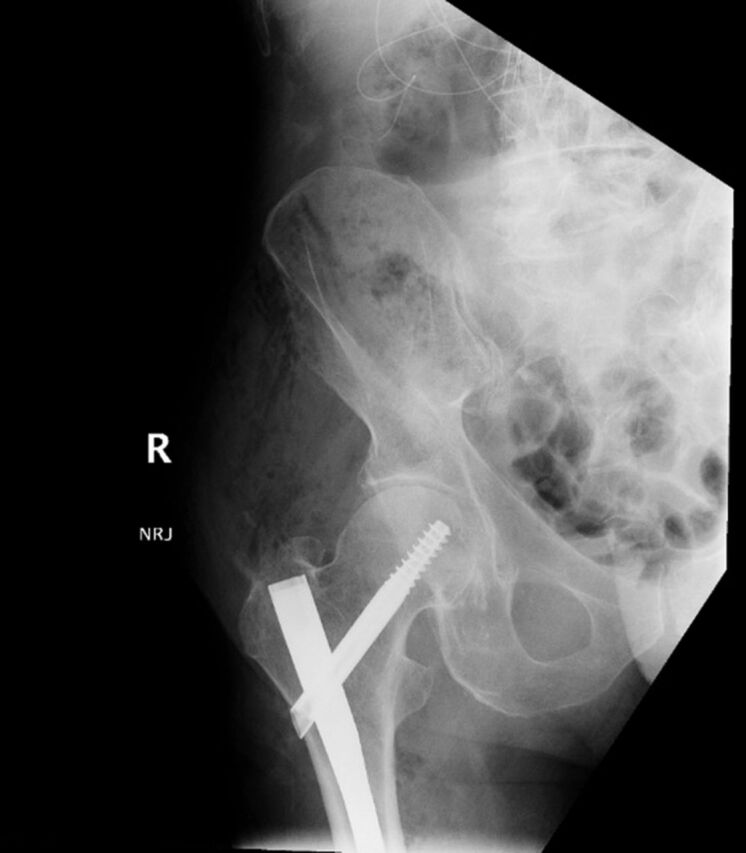
**Postoperative anteroposterior x-ray of the right femur shows a cephalomedullary nail and a 100-mm hip screw.**

The patient's preoperative laboratory workup was unremarkable; his hemoglobin (Hgb) level prior to surgery was 11.4 g/dL. Standard trochanteric-entry cephalomedullary nail fixation was performed without complication. Blood loss of 75 cc was noted intraoperatively; no excessive bleeding or drainage was noted. The patient was transported to the postanesthetic care unit in stable condition (Figure 3).

**Figure 4.**
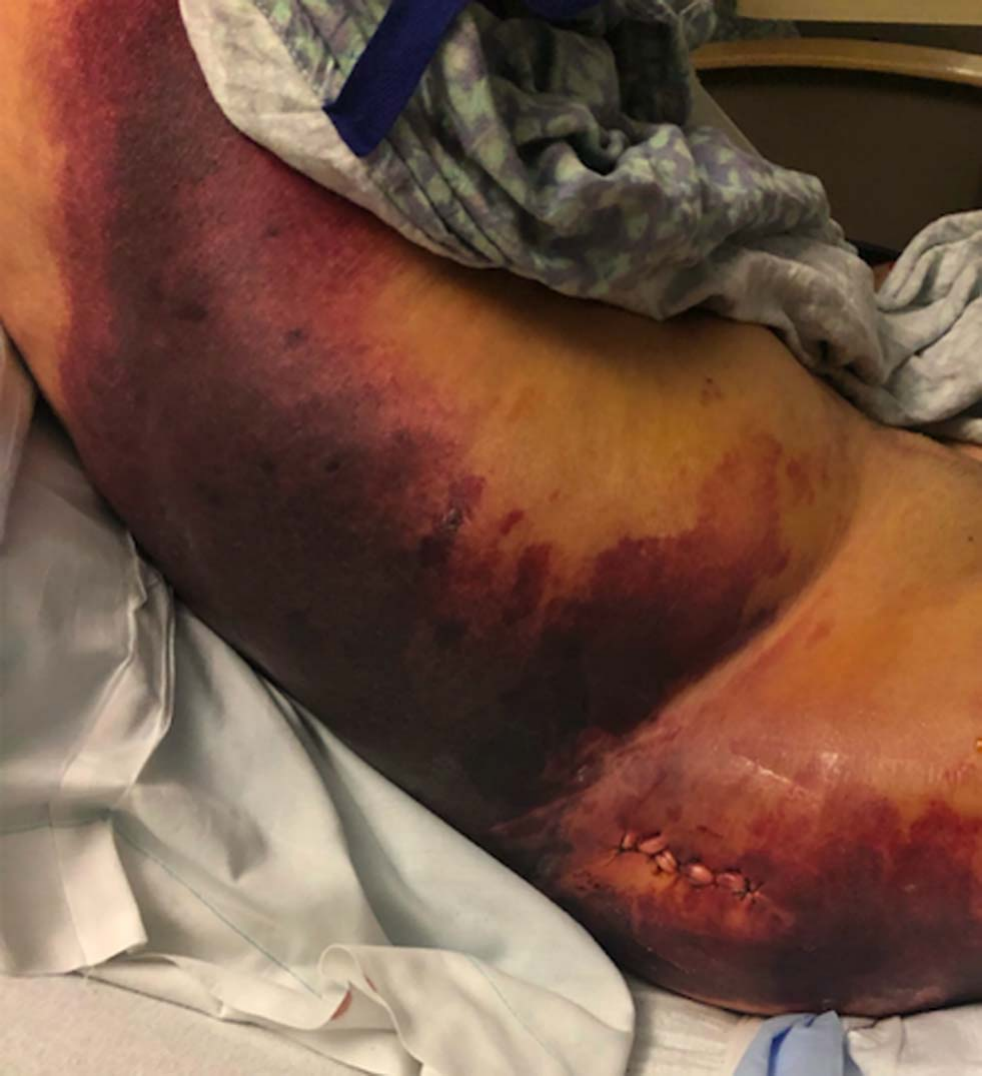
**On postoperative day 4, clinical examination revealed ecchymosis.**

**Figure 5.**
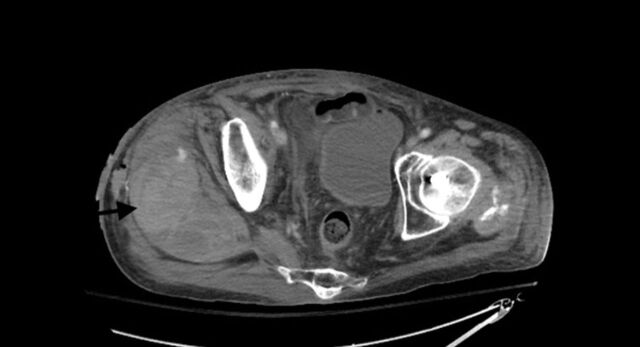
**Computed tomography with contrast of the pelvis shows a 9 × 8 × 12-cm right-sided hematoma (arrow).**

On postoperative day (POD) 1, the patient reported minimal pain, and his surgical site was clean, dry, and intact on examination. Because his Hgb level was 6.5 g/dL, he was transfused with 1 unit of packed red blood cells (PRBCs). On POD 2, the patient's Hgb level continued to decrease despite transfusion. The morning laboratory workup showed 5.0 g/dL, but the patient had no signs of bleeding on examination. On POD 2, he received 2 units of PRBCs but refused a posttransfusion Hgb check. The patient was combative and confused on POD 3 and refused the laboratory workup; physical examination findings were unchanged. On POD 4, the patient's Hgb level was 4.0 g/dL, and physical examination revealed right low back, gluteal, and thigh hematomas with severe ecchymosis (Figure 4). Computed tomography scan with contrast of the abdomen and pelvis showed a 9 × 8 × 12-cm, multiloculated, heterogenous fluid collection adjacent to the femur with active bleeding from the right superior gluteal artery (Figure 5).

**Figure 6.**
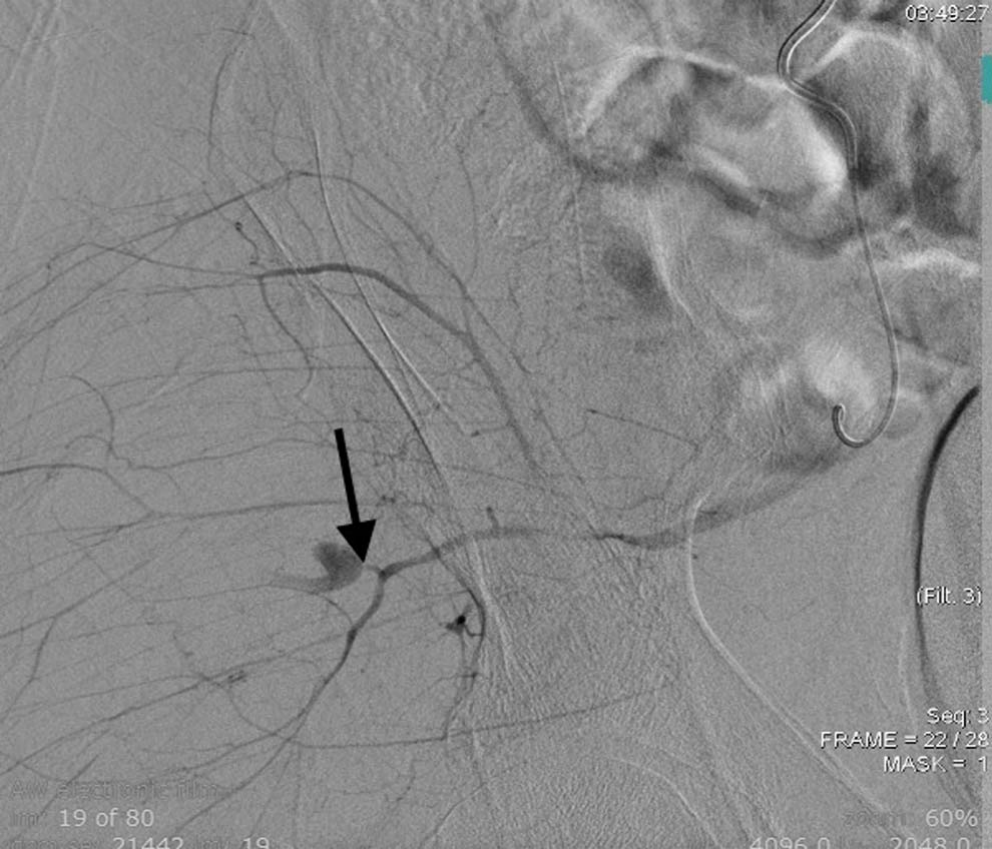
**Angiography shows active bleeding from a deep branch of the superior gluteal artery (arrow).**

The patient was treated with coil embolization on POD 5. The bleed was located in the deep branch of the right superior gluteal artery (Figure 6). The patient's Hgb level steadily increased postembolization and was 8.8 g/dL on POD 9. The patient was discharged to a skilled nursing facility on POD 9 in stable condition.

## DISCUSSION

Published reports of the occurrence and treatment of superior gluteal artery injuries after antegrade femoral nailing are scarce, perhaps because of the rarity of the complication. A thorough understanding of superior gluteal artery anatomy is critical to understand and prevent this complication.

**Figure 7.**
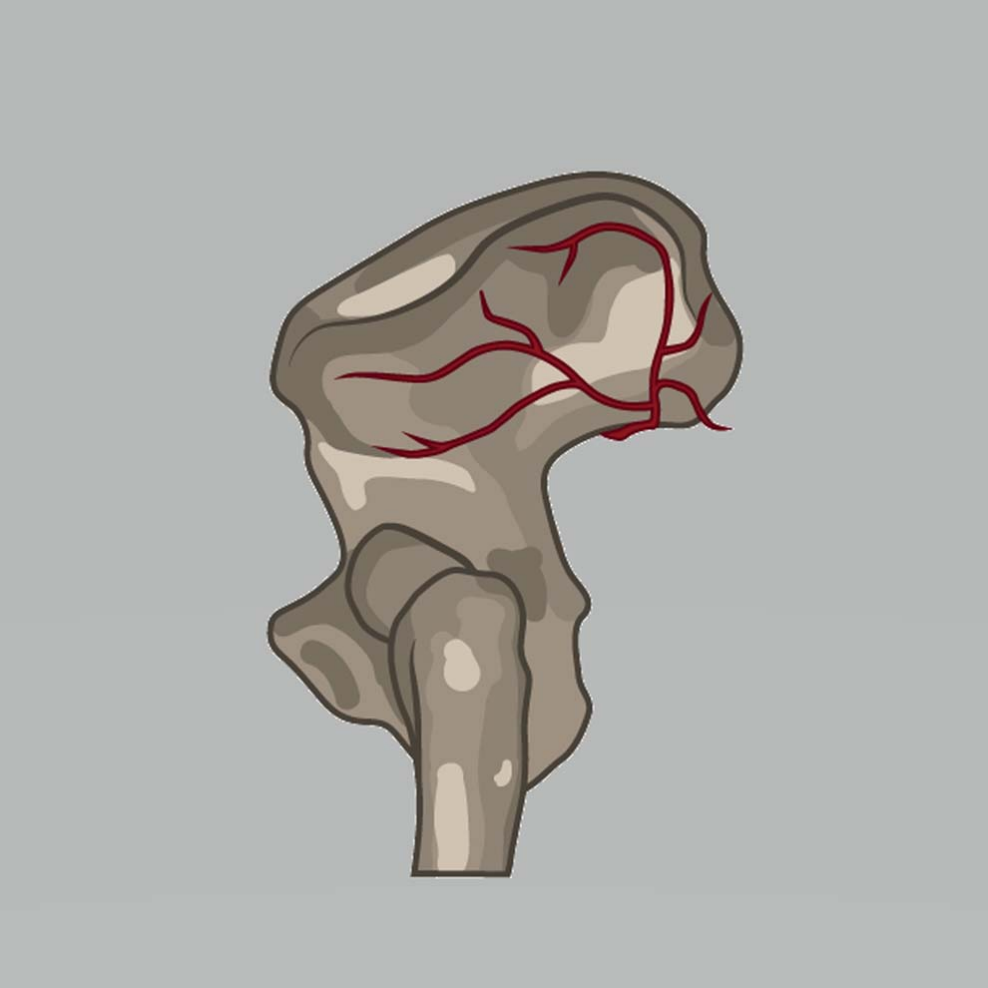
**This illustration of the superior gluteal artery shows the superficial branch coursing superiorly and posteriorly and the deep branch coursing anteriorly.**

The superior gluteal artery exits the pelvis through the greater sciatic foramen above the piriformis and then divides into a superficial and a deep branch (Figure 7). The superficial branch supplies the gluteus maximus, while the deep branch divides into superior and inferior divisions deep to the gluteus medius. The superior division courses superiorly to the anterior superior iliac spine, while the inferior division dives down toward the greater trochanter. The anatomic position can vary between patients.^10,11^ The superior gluteal artery and its branches must be visualized and avoided in extended posterior approaches to the hip.^10,11^ While visualization of these branches is necessary in posterior approaches to the hip, exposing the vessels during routine femoral nailing is unnecessary.

We found 1 published report of a superior gluteal artery bleed that occurred with antegrade femoral nail placement.^9^ The patient sustained a subtrochanteric femur fracture, was treated with a piriformis nail in standard locking fashion, had progressive anemia postoperatively, and was treated with serial transfusions. Clinical examinations showed little evidence of bleeding until POD 7, when the patient complained of severe thigh pain and hematoma. Imaging confirmed a superior gluteal artery bleed that was embolized.

The piriformis fossa starting point for femoral nailing is directly in line with the medullary canal, whereas the starting point of a trochanteric entry nail is just lateral to the medullary canal.^12,13^ Traditionally, the piriformis fossa starting point is considered to be more challenging, especially in patients who are obese.^14^ One cadaver-based study comparing the difference in soft-tissue injury with each starting point showed a higher risk of vascular injury with piriformis nails, particularly to the medial circumflex femoral artery, but no injuries to the superior gluteal artery were found.^12^ Theoretically, a more medial approach, such as a piriformis fossa approach compared to a trochanteric entry, would increase the risk of a superior gluteal artery bleed because of the proximity of the guidewire and nail to the superior gluteal artery and its branches during insertion. However, the cadaver study did not document any injuries to the superior gluteal artery with a piriformis nail in particular, although the case report from Ward et al described a bleed associated with the piriformis approach.^9,12^

Ward et al hypothesized that the patient's superior gluteal artery was injured with the guidewire when the surgeon was trying to find the proper starting point.^9^ We hypothesized that such an injury likely occurred in our case and therefore recommend extreme caution with guidewire placement and particular attention to not overmedializing the starting point. To avoid medialization of the guidewire, an incision of sufficient size and location is key to find the correct starting point for the guidewire. An incision that is too small or too close to the greater trochanter may force the guidewire medially and disrupt the proper angle for entry into the greater trochanter. Fluoroscopy is crucial to visualize the greater trochanter for guidewire placement. AP and lateral x-rays are important for determining a correct entry point, especially if the leg is rotated or abducted/adducted for reduction. Small variations from a true AP or lateral x-ray may be deceiving by giving the impression that the guidewire is at an ideal starting point, but the position may be more medial than desired.

Our patient's history of alcohol abuse may have played a role in his complication. His preoperative laboratory values were all within normal limits, including prothrombin time, partial thromboplastin time, platelet count, and liver function tests. Despite the normal laboratory values, his vessel fragility from alcohol abuse predisposed him to bleeds.

Superior gluteal artery bleeds, although rare, should be considered in antegrade femoral nail cases complicated by postoperative anemia. In both cases presented in this report, the only early sign of bleeding was continued anemia despite serial transfusions. Not until POD 4 in our case and POD 7 in the Ward et al case did either patient show signs of bleeding on examination.^9^ We recommend close examination and early imaging for patients who have undergone antegrade femoral nail placement and are experiencing progressive anemia. Early diagnosis of superior gluteal artery injury will decrease the time to embolization and thus may decrease length of stay, morbidity, and mortality.

## CONCLUSION

Superior gluteal artery bleed is a rare complication associated with cephalomedullary nail fixation for an intertrochanteric femur fracture. Placement of the guidewire requires careful consideration. A superior gluteal artery bleed can be life-threatening, so a high index of suspicion for such an injury is vital for patients with decreasing Hgb levels, regardless of their clinical signs.

## Notes

This article meets the Accreditation Council for Graduate Medical Education and the American Board of Medical Specialties Maintenance of Certification competencies for Patient Care and Medical Knowledge.
